# Clinical Efficacy of Curcumin and Vitamin E on Inflammatory-Oxidative Stress Biomarkers and Primary Symptoms of Menopause in Healthy Postmenopausal Women: A Triple-Blind Randomized Controlled Trial

**DOI:** 10.1155/2022/6339715

**Published:** 2022-06-09

**Authors:** Azizeh Farshbaf-Khalili, Alireza Ostadrahimi, Mojgan Mirghafourvand, Khatereh Ataei-Almanghadim, Sara Dousti, Amir Mehdi Iranshahi

**Affiliations:** ^1^Physical Medicine and Rehabilitation Research Centre, Aging Research Institute, Tabriz University of Medical Sciences, Tabriz, Iran; ^2^Nutrition Research Center, Tabriz University of Medical Sciences, Tabriz, Iran; ^3^Social Determinants of Health Research Center, Tabriz University of Medical Sciences, Tabriz, Iran; ^4^Student Research Center, Department of Midwifery, Faculty of Nursing and Midwifery, Tabriz University of Medical Sciences, Tabriz, Iran; ^5^Department of Midwifery, Mahabad Branch, Islamic Azad University, Mahabad, Iran; ^6^Department of Clinical Nutrition, Faculty of Nutrition and Food Sciences, Tabriz University of Medical Sciences, Tabriz, Iran

## Abstract

*Aims of the Study*. Reducing estrogen levels due to menopause activates oxidative and inflammatory processes, which causes symptoms of menopause, anxiety, and sexual dysfunction. As a suggestion, potential anti-inflammatory and antioxidant agents such as curcumin and vitamin E could be used as an effective alternative treatment due to parsimony, suitable access, and fewer side effects. Therefore, the present study was conducted to find out whether supplementation with curcumin and vitamin E affects inflammatory-oxidative stress biomarkers and primary symptoms of menopause in healthy postmenopausal women. *Methods Used to Conduct the Study*. The present study is a triple-blind parallel randomized controlled trial. Eighty-four eligible postmenopausal women aged 40 to 60 years old were randomly assigned into three groups using block randomization with an allocation ratio of 1 : 1 : 1. The curcumin group received one capsule containing 500 mg curcumin twice a day, the vitamin E group received one 500 mg capsule of vitamin E twice a day, and the placebo group took two placebo capsules containing 500 mg of microcrystalline cellulose (MCC) daily for eight weeks. Demographic and anthropometric characteristics, dietary intake, and early symptoms of menopause were collected at baseline. Serum levels of total antioxidant capacity (TAC), malondialdehyde (MDA), and high sensitivity C-reactive protein (hs-CRP) were measured at baseline and after the intervention. Intervention safety and satisfaction with the intervention were also evaluated. *Results of the Study*. Eighty-one participants completed the trial and were finally analyzed. There were no statistically significant differences in demographic characteristics and dietary intake of participants (except for vitamin C intake, *P*=0.023) between the groups at baseline. The mean ± standard deviation (SD) score of total menopause symptoms, depression, anxiety, psychological, vasomotor, and physical domains significantly decreased within all groups (*P* < 0.05). Between-group analyses indicated that decreasing the mean score of anxiety in the vitamin E group was significantly more than in the placebo group (*P*=0.026). The mean (SD) serum levels of MDA and hs-CRP significantly decreased only in the curcumin group (*P*=0.009 and *P*=0.025, respectively). Serum levels of TAC significantly increased in curcumin and vitamin E groups (*P* < 0.001 and *P*=0.006, respectively). *Conclusions Drawn from the Study and Clinical Implications*. Curcumin could improve the oxidative stress (MDA and TAC) and inflammatory (hs-CRP) biomarkers. Vitamin E may also improve the antioxidant status by increasing the TAC levels. The alleviation of anxiety in the vitamin E group was more than in the placebo group. *Clinical Trial Registration*. The trial was registered at the Iranian Registry of Clinical Trials (https://irct.ir/IRCT20131009014957N6).

## 1. Introduction

Chronic disease is a prolix condition that induces physical changes and decreases body enactment, which can not be precluded by vaccines or completely healed by medication. It has recently been considered due to the prolonged treatment period and high health costs, which are also rising [1].

Related studies indicate that persistent oxidative stress and inflammation can be effective in the pathophysiology of chronic diseases; consequently, antioxidant and anti-inflammatory agents could alleviate these conditions [1–3].

The menopausal syndrome is one of the major chronic inflammatory conditions in middle-aged women [4]. Menopause is characterized by reducing estrogen levels, which leads to a wide range of symptoms including hot flashes, breast tenderness, vaginal dryness, irregular menstruation, mood swings, atrophic vaginitis, osteoporosis, and cardiovascular disorders [5, 6]. The menopausal syndrome occurs following a decrease in the body's antioxidant potential due to the fall in estrogen levels and age increasing [7]. As a consequence, the body is exposed to oxidative stress, which induces chronic inflammation [1, 2, 7].

There are numerous therapeutic approaches to ameliorate menopausal symptoms during the estrogen-deficient phase, the most common of which is hormone replacement therapy (HRT) [8]. However, due to the possible serious side effects of HRT (particularly thromboembolic accidents, stroke, and breast cancer), the efficacy of HRT has dwindled, and there has been a rising interest in complementary and alternative medicine (CAM). CAM is a collection of various health approaches, which are not included in the customary medical treatments. Alternative medical systems, mind-body training, biologically based methods, body-based methods, and energy therapies are the main domains of CAM [9].

Dietary modifications, herbal remedies, and vitamin applications are common CAM methods used to alleviate menopause symptoms [9, 10].

Guidelines have demonstrated well-balanced energy, and a protein diet with adequate portions of dietary fiber could relieve the menopausal syndrome. On this matter, an appropriate diet accentuates the consumption of fruits, vegetables, whole grains, nuts, legumes, flaxseed, dairy products, egg, soy, prebiotics, probiotics, and omega-3 fatty acids, and departure of salt, saturated fatty acids, and dietary cholesterol [9–12].

Herbal derivatives are widely used by middle-aged women to manage menopausal symptoms due to their beneficial effects, availability, and low side effects. Herbal agents are used generally as multibotanical products; therefore, pieces of evidence supporting the efficacy of single herb formulas are less encountered [1, 9]. Curcumin is a herbal compound considered to be a common healthy treatment with notorious metabolic and safety properties [7, 13]. Curcumin is a polyphenolic component of the turmeric spice (Curcuma Longa), which exerts anticancer, anti-inflammatory, and antioxidant properties. It also exerts protective effects against cardiovascular diseases [14]. Curcumin may be beneficial for postmenopausal women in terms of lipid peroxidation-related demonstrations including diabetes, dyslipidemia, and myocardial infarction; however, there is not sufficient evidence for other inflammatory states and menopausal symptoms [15, 16].

Menopausal women usually take vitamins to reduce menopausal morbidities and mortality. Vitamins are taken from foods or commercially in pill forms during increased needs periods, such as menopause. Vitamin E, also known as a menopausal vitamin, is recently considered an estrogen alternative [9]. The vitamin E family is comprised of eight fat-soluble compounds including four tocopherols and four tocotrienols that exert antioxidant activities [17]. Few studies have evaluated the efficacy of vitamin E in terms of alleviating menopausal symptoms, the results of which are contradictory [9]. Due to the concerns regarding the safety of vitamin E in high doses (more than 1000 mg/day) and the scarcity of conclusive evidence supporting the effects of vitamin E on menopausal syndrome management, the clinical efficacy of vitamin E in this regard is suspicious [9, 18].

The number of postmenopausal women is rising instantly considering the increased life expectancy. The extended arrays of symptoms attributed to menopause gradationally deteriorate the well-being of these women and ultimately their quality of life. Thereby, maintaining the optimal health of postmenopausal women with inferior and safe methods is substantial [19, 20].

Considering that the pieces of evidence evaluating the efficacy of curcumin and vitamin E in relieving menopausal symptoms are poor and contradictory, it is necessary to conduct an exhaustive randomized clinical trial in this regard [9, 15, 16, 20].

Hence, the present study was conducted to find out whether supplementation with curcumin and vitamin E affects inflammatory-oxidative stress biomarkers implicated in the pathogenesis of menopause and primary symptoms of menopause in healthy postmenopausal women.

## 2. Materials and Methods

### 2.1. Study Design and Participants

The present study is a triple-blind parallel-arm randomized controlled trial, stratified based on body mass index (BMI), which was performed on 84 healthy postmenopausal women (40 to 60 years old) recruited from health centers of Tabriz, Iran, from June 2019 to April 2020. The primary outcomes of the present study were mean changes in serum total antioxidant capacity (TAC), serum malondialdehyde (MDA), and menopausal symptom score. The secondary outcomes included the mean change of serum high sensitivity C-reactive protein (hs-CRP).

### 2.2. Inclusion and Exclusion Criteria

Inclusion criteria included the willingness to participate in the study, having enough literacy to fill out a questionnaire or the presence of a literate person in the family, duration of menopause less than 6 years, having a minimum of 40 years and a maximum of 60 years, a minimum score of 15 and a maximum of 42 in the assessment of Greene climacteric scale, and having hot flashes. Exclusion criteria also included the use of tobacco, alcohol, and herbal medicines, the presence of stressors such as the death of close relatives in the last 6 months and job loss, the presence of gallstones and gallbladder obstruction, the presence of malignant disease, a history of surgery and recent trauma (during the last six months), acute and chronic uncontrolled inflammatory disease (including diabetes, rheumatoid arthritis), history of allergies to turmeric, use of anticoagulants (heparin, warfarin, enoxaparin, etc.), taking curcumin supplements or estrogen therapy during the 3 months before the study, and the use of special diets.

### 2.3. Sample Size Justification

The sample size was determined using G-power software (version 3.1.2) and based on the study of Panahi et al. [21] for the MDA variable considering *m*1 = 3.90, *m*2 = 3.05, sd1 = 0.06, sd2 = 0.91, two-sided test, *α* = 0.05, and power = 85% equal to 26 women; for TAC variable considering *m*1 = 3.17, *m*2 = 3.84, sd1 = 0.41, sd2 = 0.62, two-sided test, *α* = 0.05, and power = 85% equal to 13 people; and based on the study of Ataei et al. [22] for the hot flash variable considering *m*1 = 0.32, *m*2 = 7.4, sd1 = 8, sd2 = 6.6, two-sided test, *α* = 0.05, and power = 85% equal to 7 people; considering the largest sample size for MDA and calculating a 10% drop probably, the final sample size of 28 people was estimated in each group.

### 2.4. Method of Randomization and Concealment

The process of generating a random allocation sequence and allocation concealment was performed by a statistical advisor who had no clinical involvement in the study. The participants were randomly assigned into three groups using the block randomization method (block size: six and nine) and random allocation software (RAS) with an allocation ratio of 1 : 1 : 1. In order to apply the concealment in the randomization process and allocation sequence, the statistical advisor encoded unique codes on the identical boxes, which were generated by the software, and identical black, opaque, and numbered containers (in the sequence of allocation sequences) containing curcumin, vitamin E, or placebo capsules were used.

By entering each participant into the study based on the sequence produced, the boxes of 60 capsules were delivered to them based on the assigned code at baseline and four weeks later.

Besides, the research team, participants, clinical and laboratory staff, and even statisticians were blind until the end of the data analysis to ensure the accuracy of randomization, allocation, and intervention.

### 2.5. Characteristics of Supplements

Supplements and their placebos were prepared by Barij Essence Pharmaceutical Company (Kashan-Iran) with the same shape, color, scent, and size. Curcumin capsules were standardized based on 95% Tumeric root extract which contained 475 mg curcuminoid and vitamin E capsules were standardized based on 200 international units (IU) of alpha-tocopherol. Placebo capsules contain 500 mg of MCC.

### 2.6. Intervention

In the beginning, participants were asked to continue their common eating habits and physical activity during the intervention. The intervention lasted for eight weeks during which the first intervention group, i.e., the curcumin group, received one capsule containing 500 mg of curcumin twice a day, the second intervention group (vitamin E group) received one 500 mg capsule of vitamin E twice a day, and the third intervention group (placebo group) took two placebo capsules containing 500 mg of MCC daily.

The duration of intervention was divided into two periods of four weeks. Two opaque sealed envelopes, containing a box of 60 capsules (supplement or placebo) for every four weeks, were considered for each participant. Stratification was performed based on BMI (according to the BMI distribution ratio in the files available in health centers, the first nine doubled envelopes were allocated to the normal group, 36 second envelopes to the overweight group, and 39 third envelopes to the obese group).

In the beginning, one of the envelopes was delivered to each participant along with a checklist for recording the supplement use. In order to assess the compliance with the supplementation, participants were asked to refer to the health care centers every four weeks and submit a four-week supplement consumption checklist with the previous box (whether empty or full). Therefore, compliance was estimated by counting the remaining capsules. After noting how participants used their supplements and receiving the four-week consumption checklist, they were given a new envelope and checklist at the end of each period. The researchers also monitored the status of supplement consumption by telephone every week.

### 2.7. Data Collections

#### 2.7.1. Demographic Characteristics

Demographic characteristics including age, menopause age, childbirth, education status, marital status, living status, life satisfaction, occupation, family income, family members, and husband profile (including education, occupation, and smoking status) were collected using a demographic questionnaire at baseline.

#### 2.7.2. Anthropometric Assessment

At baseline, weight was measured by a lever scale (Seca, Hamburg-Germany) with a precision of 0.1 kg with minimal clothing. Height was also measured using a wall-mounted stadiometer (Seca, Hamburg-Germany) with an accuracy of 0.1 cm without shoes in a standard position (back of the head, shoulders, pelvis, and back of the legs and heel tangent to the wall). BMI was calculated as dividing weight in kilograms by height in meters squared.

#### 2.7.3. Physical Activity

Physical activity was measured by the international physical activity questionnaire-short form (IPAQ-SF). It is calculated using the following formula: MET level × minutes of activity/day × days per week. The validity and reliability of this questionnaire have been approved in 12 countries [23]. The reliability of the Persian version of this questionnaire was also confirmed in the study by BashiriMoosavi et al. [24].

#### 2.7.4. Early Symptoms of Menopause Assessment

At baseline, primary symptoms of menopause were evaluated by the Greene climacteric scale [25], the validity and reliability of which have been confirmed in Iran [26]. Participants who scored a minimum of 15 and a maximum of 42 were included in the study.

#### 2.7.5. Dietary Intake Assessment

Dietary intake was evaluated by a three-day food record questionnaire (two nonconsecutive weekdays and one weekend day) at baseline. It was analyzed in terms of total energy, macronutrients, fiber, vitamins, and minerals using Nutritionist IV software (First Databank, San Bruno, CA), modified based on Iranian foods.

#### 2.7.6. Blood Sampling and Biochemical Assessment

After twelve hours of overnight fasting, 5 ml of venous blood was collected from each participant at baseline and the end of the intervention. Serum samples were obtained from whole blood using centrifugation at 3,000 revolutions per minute (rpm) for 10 min at room temperature and immediately stored at −70°C until assay time.

Serum MDA was measured based on reaction with thiobarbituric acid (TBA), extraction with normal butanol, absorption measurement by spectrophotometry, and comparison of absorption with standard curve using Eastbiopharm kit. To measure TAC, 2,2-azino-bis-3-ethylbenzothiazoline-6-sulfonic acid (ABTS) was incubated with peroxidase (meth myoglobin) and H_2_O_2_ to produce the ABTS radical cation, which has a relatively stable green and blue color and is measured at the wavelength of 600 nanometers. The antioxidants of added sample suppressed the concentration of this colored product in proportion to their concentration and were measured with a Biorex kit. Finally, hs-CRP was assayed using Pars Azmoon kit through enhanced turbidometric method for two-point measurement with photometer based on complex formation resulting from the reaction between hs-CRP and antiserum by Abbott Alcyon 300 Biochemistry Analyzer.

### 2.8. Validity and Reliability Assessment

In this study, the validity of the questionnaire of social-demographic characteristics and checklists was determined through content and face validity. To confirm the reliability of the laboratory tests, two samples with different names were measured for five people and the correlation coefficients were confirmed.

### 2.9. Statistical Analysis

All data were analyzed using SPSS (version 24.0) (SPSS, Chicago, IL, USA). Analyses were performed based on the intention-to-treat (ITT) approach for all participants who were randomly assigned. The expectation-maximization was used for the estimation of missing values. A *P* value of less than 0.05 was considered statistically significant. Kolmogorov-Smirnov test was used to assess the normality of data distribution. Quantitative data were presented by mean (SD) and median (percentile 25- percentile 5), and qualitative data were presented by number (percent). One-way analysis of variance (ANOVA), Kruskal-Wallis, and chi-square tests were used to compare general and baseline data between the groups. Also, an independent sample Kruskal-Wallis test was used to compare satisfaction with medication between the groups. Analysis of covariance (ANCOVA) was employed to compare the differences between the groups after intervention adjusted for baseline measures and confounding factors at the end of the intervention. The paired *t*-test was also applied to compare the differences within the groups in parametric variables.

### 2.10. Ethical Considerations

The researchers adhered to all the standards of the Helsinki declaration, and informed consent was obtained from all of the participants. The trial was approved by the Ethics Committee of Tabriz University of Medical Sciences with ID code IR.TBZMED.REC.1399.035 and was registered at the Iranian Registry of Clinical Trials (https://irct.ir/IRCT20131009014957N6).

### 2.11. Safety and Satisfaction Assessment

For safety assessment and control of side effects, patients were monitored before, during, and after the intervention based on a checklist containing symptoms such as nausea, vomiting, stomachache, diarrhea, hypertension, allergic reactions, and open question about any other side effects.

Satisfaction with supplementation was evaluated by an item at the end of the study, which was scored from 1 to 5 based on the Likert scale, including completely dissatisfied, somewhat dissatisfied, neither satisfied nor dissatisfied, somewhat satisfied, and completely satisfied.

## 3. Results

### 3.1. General Characteristics

**Figure 1:**
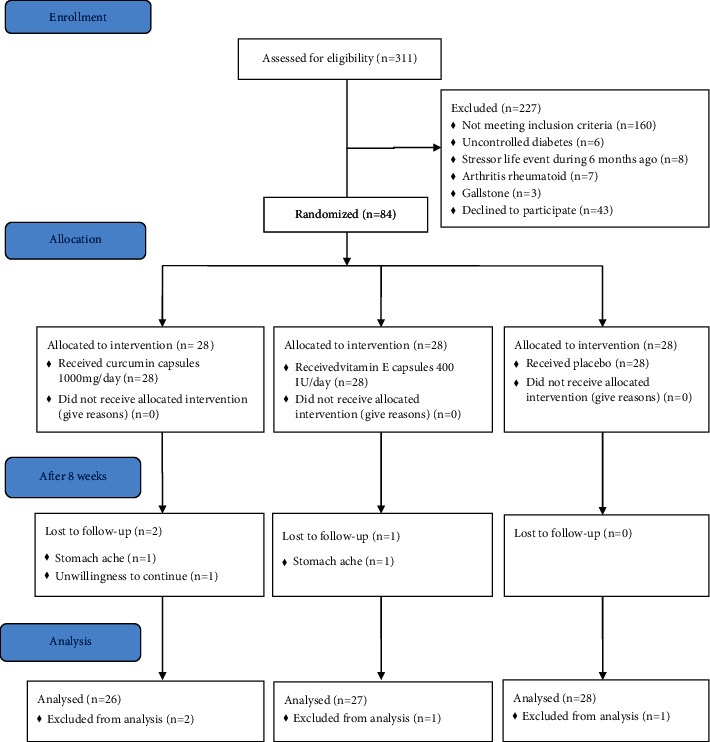
Flow diagram of participants' summary.

Participants included 84 healthy postmenopausal women (*n* = 28 in each group). The mean (SD) of age, menopausal age, and BMI of participants was 52.3 (2.9), 49.3 (2.9), and 29.2 (7.2), respectively. Finally, 81 participants (*n* = 26, *n* = 27, and *n* = 28 in curcumin, vitamin E, and placebo groups, respectively) completed the trial and were analyzed. In total, two people in the curcumin group (one person due to stomach ache and one person due to unwillingness to continue) and one person in the vitamin E group due to stomachache withdrew from the study (Figure 1). Capsule counts indicated that the participants' compliance with the intervention was over 90% in all groups.

There were no significant differences in the demographic characteristics of participants and total physical activity between the study groups (*P* > 0.05) (Table 1).

### 3.2. Dietary Intake

The mean (SD) intakes of energy, carbohydrate, protein, and total fat were 2160 (380) kcal/day, 324 (53) g/day, 59 (14) g/day, and 74 (17) g/day, respectively. The mean (SD) intake of micronutrients such as saturated fatty acids (SFAs), polyunsaturated fatty acids (PUFAs), monounsaturated fatty acids (MUFAs), total fiber, vitamin E, vitamin C, vitamin A, and vitamin D was 36 (5) g/day, 22 (4.5) g/day, 32 (6) g/day, 64 (17) g/day, 19 (3) mg/day, 157 (34) mg/day, 615 (117) mg/day, and 1.8 (0.6) *µ*g/day, respectively. The mean (SD) intake of minerals such as selenium and zinc was 101 (24) *μ*g/day and 10 (2) mg/day, respectively. There was a statistically significant difference in vitamin C intake between the groups at baseline (*P*=0.023). Therefore, it was considered a confounder and adjusted during data analysis. Other baseline characteristics were the same in the intervention and placebo groups (Table 2). The mean (SD) intake of curcumin was 567.4 (374.2) mg/day among participants.

### 3.3. Menopausal Symptoms

The mean (SD) score of total menopause symptoms, depression, anxiety, psychological, physical, and vasomotor domains significantly decreased within all groups. However, the between-group analyses indicated significant differences in terms of total menopause symptoms (*P*=0.048) and anxiety dimension (*P*=0.021). The decrease in the mean score of anxiety in the vitamin E group was significantly more than the placebo group (*P*=0.026) as well as improvement of total menopause symptoms in the group vitamin E was more than the other groups (Table 3).

### 3.4. Serum Levels of MDA, TAC, and Hs-CRP

The mean (SD) serum levels of MDA [MD (95% CI): −0.5 nmol/mL (−0.8 to −0.1); *P*=0.009] and hs-CRP [MD (95% CI): −0.5 mg/L (−0.8 to −0.2); *P*=0.025] significantly decreased only in the curcumin group. Serum levels of TAC significantly increased in curcumin [MD (95% CI): 0.2 mmol/L (0.1 to 0.35); *P* < 0.001] and vitamin E groups [MD (95% CI): 0.1 mmol/L (0.01 to 0.2); *P*=0.006]. However, there were no significant differences between groups in the serum biomarkers (Table 4).

### 3.5. Satisfaction with Supplementation

At the end of the interventions, 88.5% of the participants in the curcumin, 92.6% in the vitamin E, and 89.3% in the placebo groups were completely satisfied or satisfied with supplementation. The rate of dissatisfaction was 11.5%, 7.4%, and 10.7% in the curcumin, vitamin E, and placebo groups, respectively (Table 5).

### 3.6. Adverse Events

A total of 17 participants (*n* = 2, *n* = 12, and *n* = 3 in curcumin, vitamin E, and placebo groups, respectively) reported side effects due to supplement use. Reported events included stomachache (two people in the placebo group, five people in the vitamin E group, and one person in the curcumin group), hypertension (one person in each of the curcumin and vitamin E groups), breast engorgement (one person in each of the vitamin E and placebo groups), headache, vomiting, diarrhea, allergy, and skin rash (one person in the vitamin E group). The severity of the reported side effects was mild to moderate and occurred at the beginning of supplementation and not continued except for stomach ache which led to dropping out in two women.

## 4. Discussion

### 4.1. Rationale

Previous studies have shown that curcumin and vitamin E have antioxidant and anti-inflammatory effects; however, these effects have not been comprehensively investigated in healthy postmenopausal women. To the best of our knowledge, our study is the first report conducted to compare the effects of curcumin and vitamin E supplements on TAC, MDA, and hs-CRP in healthy postmenopausal women.

The present study demonstrated that curcumin and vitamin E supplementation could improve menopause symptoms. The mean (SD) score of total menopause symptoms, depression, anxiety, and psychological and vasomotor domains significantly decreased within all groups, except for the sexual domain. The between-group analyses indicated that decreasing the mean (SD) score of anxiety in the vitamin E group was significantly more than in the placebo group. On the other hand, the mean (SD) score of total menopause symptoms in the vitamin E group decreased more than in the other two groups. The mean (SD) serum levels of MDA and hs-CRP significantly decreased only in the curcumin group. Serum levels of TAC significantly increased in curcumin and vitamin E groups; however, the between-group difference was not significant.

### 4.2. Possible Mechanisms

Recent studies have demonstrated that inflammation and oxidative stress are involved in the pathogenesis of menopausal symptoms such as hot flashes or night sweats [1, 2].

Curcumin has been shown to implicate in biological functions regulated by microribonucleic acids (micro-RNAs) including differentiation, development, proliferation, and oxidative stress [27]. It has also been demonstrated to suppress inflammation by reducing the expression of the nuclear factor kappa B (NF-*κ*B), inhibiting reactive oxygen species- (ROS-) generating enzymes such as cyclooxygenase, lipoxygenase, and nitric oxide synthases, and inhibiting the production of inflammatory cytokines such as tumor necrosis factor-alpha (TNF-*α*), interleukin (IL)-1, IL-6, IL-8, and IL-12 [28–30]. Curcumin inhibits the production of oxygen free radicals by decreasing MDA concentration, upregulating gene expression of manganese superoxide dismutase (SOD), copper/zinc-SOD, glutathione peroxidase- (GPX-) 4 and GPX-1, and increasing catalase (CAT) and SOD activity [27].

Vitamin E has been known as a potent immunomodulator, which is involved in cell membrane integrity, inflammation, signal transduction, and cell cycle [31]. It appears to decline the inflammatory response by inhibiting the NF-*κ*B pathway, reducing the activity of inflammatory cytokines including TNF-*α*, IL-6, and IL-1*β*, interfering with the enzymatic activity of cyclooxygenase 2, and decreasing the production of inflammatory mediators such as prostaglandin E_2_ [31, 32]. Vitamin E also acts as a free radical scavenger and protects the cell membrane against oxidation and pathogens [31]. The antioxidant properties of vitamin E are induced by the upregulation of the antioxidant enzymes gene through the regulation of the nuclear factor-erythroid 2-related factor 2 (NRF2) transcription factor [32].

### 4.3. The Effects of Curcumin and Vitamin E on Early Menopausal Symptoms

Symptoms of vasomotor include hot flashes and night sweats, which are one of the most annoying symptoms of menopause [33]. Vasomotor symptoms not only reduce the quality of life due to the cessation of estrogen production but are also a risk factor for cardiovascular disease [34].

In a clinical trial conducted by Akazawa and Maeda, the effects of curcumin and exercise on vascular endothelial function in postmenopausal women were evaluated. In this study, 32 postmenopausal women were divided into three groups (10 participants in the control group and 11 participants in each of the curcumin and the exercise groups), during which the curcumin group received a total of 6 tablets of 150 mg curcumin for 8 weeks daily. Consistent with our study, the results showed that regular consumption of curcumin or regular aerobic exercise helps to improve vascular function [35].

Ziaei evaluated the efficacy of vitamin E on hot flashes in 51 postmenopausal women in a clinical trial. Participants received a placebo for 4 weeks and then received vitamin E 400 IU/day for 4 weeks after one week of washout. There was a significant decrease in the frequency and severity of hot flashes in vitamin E treatment compared to placebo [36]. The same groups of women received both placebo and vitamin E treatments, and despite a similar dose to our study and a shorter duration of intervention with vitamin E, the difference between the main treatment and placebo was significant, unlike our study. Regarding the changes in the frequency of hot flashes based on the temperature change caused over time, the existence of two different treatment groups is recommended to evaluate the difference between these two studies.

Depression and anxiety are among the main psychological symptoms related to sex hormone deprivation in postmenopausal women, which cooccur due to estrogen deficiency status [22, 37].

Da Silva Morrone et al. focused on the effects of curcumin in alleviating changes in behavior and oxidative stress in the frontal cortex, hippocampus, and striatum of ovariectomized mice [38]. Curcumin treatment for 30 days reduced anxiety and oxidative stress in various brain conditions in oophorectomized mice. A double-blind crossover clinical trial was conducted by Esmaily Ghayour-Mobarhan to measure the effects of curcumin on anxiety and depression in 30 obese adults [39]. Participants received 1 g of curcumin per day for 30 days, and placebo was prescribed after a two-week of washout. The mean anxiety score decreased after treatment with curcumin; however, curcumin supplementation had no significant effect on depression scores. In our study, curcumin reduced anxiety and depression but was not significant compared with placebo. On the other hand, the participants in our study were postmenopausal women.

In a study by Gautam on 80 participants aged 20 to 60 years old with generalized anxiety disorder and depression, supplementation with vitamins A, E, and C for 6 weeks significantly reduced anxiety and depression. There was no control group in this study and the results were similar to the present study [40].

In a clinical trial conducted by Ataei-Almanghadim, 93 postmenopausal women were equally assigned into three groups of curcumin, vitamin E, and placebo and received the same dose as in the current study for 8 weeks. Despite using different tools from the current study to assess the anxiety and sexual function, the total score of menopause, anxiety, and sexual function improved significantly in all three groups over time; however, there was no significant difference between the two groups. In the present study, the anxiety score in the vitamin E group was significantly reduced compared to the placebo, which could be due to differences in the target population and cultural level, and as a result, there was a possible difference in the exact adherence to medication use and utilization of different tools. Regarding the efficacy of the placebo, a study that examined a drug with a placebo showed that the psychological effects of using a placebo may lead to positive effects [22]. In this study, the psychological effects of the intervention in the placebo group may have led to a significant reduction in the results. However, a cross-sectional clinical trial is recommended for further and more detailed examination.

### 4.4. The Effects of Curcumin and Vitamin E on TAC, MDA, and Hs-CRP Levels

TAC indicates the total antioxidant capacity of the blood, the low level of which indicates the presence of oxidative stress [41]. Menopause, obesity, decreased physical activity, unhealthy habits, and diets are the most important factors in increasing serum CRP, insofar as some researchers have even considered it to be the most important predictor of inflammatory diseases [25]. Peroxidation or oxidation reactions take place when lipids are exposed to oxygen, during which peroxide and then MDA are produced. By measuring MDA, the extent of fat peroxidation can be determined [42].

In a study by Judaki et al., treatment with curcumin with 700 mg oral turmeric tablets three times a day for 8 weeks, combined with triple therapy of omeprazole, amoxicillin, and metronidazole in chronic gastritis induced by *Helicobacter pylori* infection, significantly increased TAC and GPX and MDA reduction of gastric mucus after the intervention compared with baseline and triple therapy [43]. In another study by Santos-Parker in healthy middle-aged and older women, 12 weeks of curcumin supplementation (2000 mg/day Longvida®; *n* = 20) reduces resistance to artery endothelial function by increasing the bioavailability of vascular nitric oxide and reducing oxidative stress [44], which are consistent with our study but the population of the study is different.

Consistent with our study, the effects of vitamin E supplementation (*α*-tocopherol, 300 mg/day) and/or low-volume exercise for 12 weeks in postmenopausal women were evaluated in a study; serum biological antioxidant potential concentrations in both groups (vitamin E and exercise groups) were significantly higher than baseline after 12 weeks [45]. In a study by Jamilian et al. aimed at examining the effects of supplementation with 400 IU of vitamin E along with 1000 mg of omega-3 fatty acids on oxidative stress indices and inflammatory markers in women with gestational diabetes, it was found that vitamin E can lead to increase TAC and nitric oxide levels and decreases MDA levels compared with placebo [46]. Despite the similarity in dose and duration of treatment with our study, the reduction of MDA in the vitamin E group in our study was not significant, and the mean difference between the treatment and placebo groups was not also significant. This difference could be due to omega-3 supplementation.

Regarding the inflammatory markers, Martínez et al. concluded that the combination of hydroxytyrosol, omega-3 fatty acids, and curcumin reduced inflammation in patients with musculoskeletal symptoms due to aromatase, which was shown to decrease CRP levels and reduce pain [47]. In a study by Carr in 75 postmenopausal women aged 40 to 65 years old, vitamin E combined with estrogen replacement therapy reduced CRP levels but had no effect alone; the results are aligned with the present study [48].

### 4.5. Limitations and Suggestions

The short duration of intervention may lead to insignificant efficacy of curcumin and vitamin E compared to placebo on the total score of menopausal symptoms and most domains. It is worthwhile to increase the duration and the dose of the intervention according to the safety and to use more absorbable forms in future studies. The limited number of participants and small sample size was another limitation; we did not measure the probable adverse outcomes for lipid profile in this study; therefore, we suggest evaluating lipid profile in complementary studies. Participants' answers were considered correct and no exact information is available on their honesty in answering the questions. A crossover clinical trial can be helpful in completing the results of this study. It is also recommended evaluating the synergistic effects of concurrent supplementation of vitamin E and curcumin in future studies.

## 5. Conclusion

The present study showed that in the first years of menopause, curcumin supplementation could improve oxidative stress (TAC and MDA) and inflammatory (hs-CRP) indices; vitamin E supplementation may also improve antioxidant status by increasing the TAC levels. There was a significant difference between the groups in terms of the total score of menopausal symptoms. The alleviation of anxiety score in the vitamin E group was more than in the placebo group. In other domains, significant improvement occurred in all groups compared to the baseline (except for the sexual domain); however, there was no significant difference between the curcumin and vitamin E groups with the placebo group. The side effects were mild and were reported for early consumption.

## Figures and Tables

**Table 1:** Baseline characteristics of participants among the study groups.

Variable	Curcumin (*n* = 26)	Vitamin E (*n* = 27)	Placebo (*n* = 28)	*P* value
Age (year)^†^	52.0 (2.5)	52.5 (3.2)	52.1 (2.7)	0.764^*∗*^
Menopause age (year)^†^	49.3 (2.2)	49.6 (2.8)	48.6 (3.5)	0.358^*∗*^
Childbirth^†^	2.5 (1.0)	2.8 (0.9)	2.6 (1.0)	0.646^*∗*^
Family members^†^	1.6 (0.5)	1.5 (0.6)	1.4 (0.5)	0.235^*∗*^
Weight (kg)^†^	72.3 (8.8)	71.8 (10.9)	74.0 (10.4)	0.710^*∗*^
BMI (kg/m^2^)^†^	30.5 (4.6)	29.9 (4.4)	30.1 (3.7)	0.880^*∗*^
Age (year)^†^	52.0 (2.5)	52.5 (3.2)	52.1 (2.7)	0.764^*∗*^
Total physical activity (MET‑min.week)^±^	396.6 (336.6–815.5)	442.0 (326.5–1026.5)	386.5 (340.5–864.5)	0.588^*∗∗∗∗*^
Marriage status^‡^	Single and widow	5 (17.9)	3 (10.7)	3 (10.7)
	Married	23 (82.1)	25 (89.3)	25 (89.3)
Educational level^‡^	Illiterate and primary	13 (46.4)	13 (46.4)	13 (46.4)
	Secondary and high school	6 (21.4)	11 (39.3)	8 (28.6)
	Diploma and more	9 (32.1)	4 (14.3)	7 (25.0)
Occupation^‡^	Housewife	27 (96.4)	26 (92.9)	28 (100.0)
	Occupied andretired	1 (3.6)	2 (7.1)	0 (0.0)
Husband education^‡^	Illiterate andprimary	2 (7.4)	8 (28.6)	8 (28.6)
	Secondary andhigh school	12 (44.4)	10 (39.3)	7 (25.0)
	Diploma andmore	14 (50.0)	9 (33.3)	13 (46.4)
Husband occupation^‡^	Self-occupied	8 (29.6)	13 (46.4)	16 (57.1)
	Employee	5 (17.9)	3 (10.7)	4 (14.3)
	Retired	13 (46.4)	10 (37.0)	7 (25.0)
	Worker	2 (7.1)	2 (7.1)	1 (3.6)
Family income^‡^	Sufficient	13 (46.4)	11 (40.7)	15 (53.6)
	Less than sufficient	15 (53.6)	16 (59.3)	13 (46.4)
Life satisfaction^‡^	Yes	26 (92.9)	24 (92.3)	22 (84.6)
	No	2 (7.1)	2 (7.7)	4 (15.4)
Smoker husband^‡^	Yes	7 (25.0)	7 (25.0)	8 (28.6)
	No	21 (75.5)	21 (75.5)	20 (71.4)
Living with husband family^‡^	Yes	1 (3.6)	0 (0.0)	1 (3.6)
	No	27 (96.4)	28 (100.0)	27 (96.4)

BMI: body mass index, ^†^mean (SD), ^±^median (p25–p75), ^‡^number (percent); ^*∗*^one-way ANOVA, ^*∗∗*^chi-square, ^*∗∗∗*^ linear by linear chi-square, ^*∗∗∗∗*^Kruskal-Wallis.

**Table 2:** Comparison of dietary intake between participants among the study groups.

Variable	Curcumin (*n* = 26)	Vitamin E (*n* = 27)	Placebo (*n* = 28)	*P* value
Energy (kcal/day)^†^	2006.45 (532.8)	2174.6 (268.9)	2291.0 (345.1)	0.815^*∗*^
Carbohydrate (g/day)^†^	280.8 (70.1)	328.1 (38.1)	363.5 (49.7)	0.555^*∗*^
Protein (g/day)^†^	54.27 (12.9)	58.1 (12.2)	63.7 (17.7)	0.572^*∗*^
Total fat (g/day)^†^	67.3 (24.8)	61.7 (12.8)	63.1 (13.8)	0.974^*∗*^
SFA (g/day)^†^	28.9 (7.5)	23.0 (3.0)	26.4 (4.6)	0.740^*∗*^
PUFA (g/day)^†^	22.0 (6.3)	23.8 (3.8)	21.3 (3.4)	0.923^*∗*^
MUFA (g/day)^†^	33.6 (9.2)	31.7 (5.1)	30.9 (4.4)	0.959^*∗*^
Total fiber (g/day)^†^	43.1 (6.4)	54.6 (7.4)	50.9 (5.6)	0.522^*∗*^
Vitamin E (mg/day)^†^	16.7 (3.4)	22.1 (5.5)	17.7 (2.3)	0.584^*∗*^
Vitamin C (mg/day)^†^	129.7 (21.1)	146.6 (35.7)	193.5 (46.7)	0.023^*∗*^
Vitamin A (mg/day)^†^	569.7 (109.3)	490.7 (39.9)	786.1 (203.1)	0.051^*∗*^
Vitamin D (*μ*g/day)^†^	1.7 (0.87)	1.6 (0.2)	2.1 (0.63)	0.820^*∗*^
Selenium (*μ*g/day)^†^	92.5 (31.3)	99.2 (15.1)	111.3 (25.3)	0.826^*∗*^
Zinc (mg/day)^†^	9.1 (2.1)	8.8 (1.8)	11.2 (2.4)	0.451^*∗*^
Turmeric (mg/day)	533.6 (454.5)	576.5 (322.5)	592.2 (345.5)	0.688^*∗*^

SFA: saturated fatty acid, PUFA: polyunsaturated fatty acid, MUFA: monounsaturated fatty acid; ^†^Mean (SD); ^*∗*^one-way ANOVA.

**Table 3:** The score of menopause symptoms among the study groups.

Menopause symptoms	Curcumin (*n* = 26)	Vitamin E (*n* = 27)	Placebo (*n* = 28)	*P* value
*Anxiety (0–18)*				
Baseline^†^	6.4 (2.6)	6.9 (2.3)	6.4 (1.9)	0.680^*∗*^
After 8 weeks^†^	5.7 (2.6)	5.1 (2.4)	5.8 (1.8)	0.021^*∗∗*^
MD (95% CI)	−0.8 (−1.5 to −0.01)	−1.8 (−2.5 to −1.2)	−0.6 (−1.1 to −0.1)	
*PP* value	0.046^*∗∗∗*^	<0.001^*∗∗∗*^	0.032^*∗∗∗*^	
*Depression (0–15)*				
Baseline^†^	6.2 (2.8)	5.0 (2.3)	5.1 (3.0)	0.084^*∗*^
After 8 weeks^†^	4.7 (2.9)	3.6 (2.4)	3.7 (2.5)	0.923^*∗∗*^
MD (95% CI)	−1.5 (−2.5 to −0.5)	−1.4 (−2.0 to −0.8)	−1.4 (−2.0 to −0.94)	
*PP* value	0.006^*∗∗∗*^	0.001^*∗∗∗*^	<0.001^*∗∗∗*^	
*Psychological (0–33)*				
Baseline^†^	12.6 (4.7)	11.9 (3.6)	11.5 (4.3)	0.455^*∗*^
After 8 weeks^†^	10.4 (4.9)	8.6 (3.9)	9.4 (3.9)	0.058^*∗∗*^
MD (95% CI)	−2.3 (−3.9 to −0.6)	−3.2 (−4.2 to −2.3)	−2.0 (−2.8 to −1.2)	
*PP* value	0.001^*∗∗∗*^	<0.001^*∗∗∗*^	<0.001^*∗∗∗*^	
*Physical (0–21)*				
Baseline^†^	5.2 (1.5)	5.6 (1.9)	5.2 (1.9)	0.358^*∗*^
After 8 weeks^†^	4.1 (1.82)	3.9 (1.3)	4.2 (2.1)	0.107^*∗∗*^
MD (95% CI)	−1.1 (−1.8 to −0.4)	−1.7 (−2.5 to −0.8)	−1. 0 (−1.5 to −0.5)	
*PP* value	<0.001^*∗∗∗*^	>0.001^*∗∗∗*^	0.001^*∗∗∗*^	
*Vasomotor (0–6)*				
Baseline^†^	4.4 (1.0)	4.0 (1.9)	4.7 (1.1)	0.746^*∗*^
After 8 weeks^†^	2.9 (1.6)	2.1 (1.7)	2.8 (1.7)	0.551^*∗∗*^
MD (95% CI)	−1.5 (−2.2 to −0.8)	−1.8 (−2.6 to −1.1)	−1.9 (−2. 4 to −1.4)	
*PP* value	<0.001^*∗∗∗*^	<0.001^*∗∗∗*^	<0.001^*∗∗∗*^	
*Sexual (0–3)*				
Baseline^†^	1.4 (0.9)	1.9 (0.8)	1.2 (0.9)	0.467^*∗*^
After 8 weeks^†^	1.4 (0.9)	1.7 (1.0)	1.1 (0.9)	0.737^*∗∗*^
MD (95% CI)	0.0 (−0.4 to 0.4)	−0.3 (−0.6 to 0.1)	−0.05 (−0.15 to 0.05)	
*PP* value	1.00^*∗∗∗*^	0.135^*∗∗∗*^	0.330^*∗∗∗*^	
*Total score (0–63)*				
Baseline^†^	23.6 (5.9)	23.4 (3.6)	22.5 (4.6)	0.949^*∗*^
After 8 weeks^†^	18.8 (7.2)	16.4 (5.1)	17.5 (5.0)	0.048^*∗∗*^
MD (95% CI)	−4.8 (−7.4 to −2.1)	−7.0 (−8.8 to −5.2)	−5.0 (−6.5 to −3.5)	
*PP* value	0.001^*∗∗∗*^	<0.001^*∗∗∗*^	<0.001^*∗∗∗*^	

MD: mean difference, CI: confidence interval; ^†^Mean (SD); ^*∗*^one-way ANOVA, ^*∗∗*^analysis of covariance adjusted for baseline measures and confounding factors (vitamin C intake), ^*∗∗∗*^paired-samples *t*-test.

**Table 4:** Serum levels of MDA, TAC, and hs-CRP among the study groups.

Variable	Curcumin (*n* = 26)	Vitamin E (*n* = 27)	Placebo (*n* = 28)	*P* value _E/P_^‡^
*MDA (nmol/mL)*				
Baseline^†^	2.4 (0.9)	2.4 (0.9)	2.3 (1.29)	0.920^*∗*^
After 8 weeks^†^	1.9 (0.6)	2.2 (0.7)	2.1 (1.0)	0.993^*∗∗*^
MD (95% CI)	−0.5 (−0.8 to −0.1)	−0.2 (−0.9 to 0.40)	−0.2 (−0.6 to 0.1)	
*PP* value	0.009^*∗∗∗*^	0.195^*∗∗∗*^	0.230^*∗∗∗*^	
*TAC (mmol/L)*				
Baseline^†^	1.3 (0.3)	1.3 (0.20)	1.5 (0.3)	0.820^*∗*^
After 8 weeks^†^	1. 5 (0.3)	1.4 (0.20)	1.6 (0.3)	0.997^*∗∗*^
MD (95% CI)	0.2 (0.1 to 0.3)	0.1 (0.01 to 0.2)	0.05 (−0.01 to 0.1)	
*PP* value	<0.001^*∗∗∗*^	0.006^*∗∗∗*^	0.118^*∗∗∗*^	
*hs-CRP (mg/L)*				
Baseline^†^	5.1 (3. 4)	4.9 (2.3)	5.1 (3.3)	0.998^*∗*^
After 8 weeks^†^	4.6 (2.6)	4.8 (2.3)	4.8 (3.3)	0.861^*∗∗*^
MD (95% CI)	−0.5 (−0.8 to −0.2)	−0.1 (−0.7 to 0.5)	−0.3 (−0.7 to 0.1)	
*PP* value	0.025^*∗∗∗*^	0.718^*∗∗∗*^	0.219^*∗∗∗*^	

MDA: malondialdehyde, TAC: total antioxidant capacity, hs-CRP: high sensitivity C-reactive protein, MD: mean difference, CI: confidence interval; ^†^mean (SD), *P*^‡^*P* value _E/P_: *PP* value of pairwise comparison between vitamin E and placebo groups, *P*^§^*P* value _C/P_: *PP* value of pairwise comparison between curcumin and placebo groups; ^*∗*^one-way ANOVA by Tukey, ^*∗∗*^analysis of covariance adjusted for baseline measures and confounding factors (vitamin C intake), ^*∗∗∗*^paired-samples *t*-test.

**Table 5:** Satisfaction with supplementation among the study groups.

Satisfaction	Curcumin (*n* = 26)	Vitamin E (*n* = 27)	Placebo (*n* = 28)
Completely satisfied^†^	21 (80.8)	23 (85.2)	25 (89.3)
Satisfied^†^	2 (7.7)	2 (7.4)	0 (0.0)
Neither dissatisfied nor satisfied^†^	0 (0.0)	0 (0.0)	0 (0.0)
Dissatisfied^†^	3 (11.5)	2 (7.4)	3 (10.7)
Completely dissatisfied^†^	0 (0.0)	0 (0.0)	0 (0.0)

^†^Number (percent).

## Data Availability

The data that support the findings of this study are available from the corresponding authors upon reasonable request.

## Abbreviations

ABTS: 2,2-azino-bis-3-ethylbenzothiazoline-6-sulfonic acid
ANCOVA: Analysis of covariance
ANOVA: One-way analysis of variance
BMI: Body mass index
CAM: Complementary and alternative medicine
CAT: Catalase
GPX: Glutathione peroxidase
HRT: Hormone replacement therapy
Hs-CRP: High sensitivity C-reactive protein
IL: Interleukin
IPAQ-SF: International physical activity questionnaire-short form
ITT: Intention-to-treat
IU: International units
MCC: Microcrystalline cellulose
MDA: Malondialdehyde
Micro-RNAs: Microribonucleic acids
MUFAs: Monounsaturated fatty acids
NF-*κ*B: Nuclear factor-kappa B
NRF2: Nuclear factor-erythroid 2-related factor 2
PUFAs: Polyunsaturated fatty acids
RAS: Random allocation software
ROS: Reactive oxygen species
RPM: Revolutions per minute
SD: Standard deviation
SOD: Superoxide dismutase
SFAs: Saturated fatty acids
TAC: Antioxidant capacity
TBA: Thiobarbituric acid
TNF-*α*: Tumor necrosis factor-alpha.

## Additional Points

The following is already known about this subject. (i) Reducing estrogen levels in the early stages of menopause leads to numerous symptoms as well as oxidative stress. (ii) There are numerous therapeutic approaches with severe side effects suggested for estrogen deficiency during menopause. Therefore, it may be better to use healthier treatments with fewer side effects. (iii) Curcumin is one of these complementary therapies, which exerts anticancer, anti-inflammatory, and antioxidant properties. (iv) Vitamin E is another complementary therapy, which could reduce oxidative stress reactions. This study adds the following. (i) Curcumin could improve the oxidative stress (MDA and TAC) and inflammatory (hs-CRP) biomarkers in healthy postmenopausal women. (ii) Vitamin E may also improve the antioxidant status by increasing the TAC levels in healthy postmenopausal women. (iii) The alleviation of anxiety in the vitamin E group was more than in the placebo group. (iv) Significant improvement of menopausal symptoms occurred in all groups compared to the baseline (except for the sexual domain).
